# Taxonomic revision and phylogenetic position of the flying squirrel genus *Biswamoyopterus* (Mammalia, Rodentia, Sciuridae, Pteromyini) on the northern Indo-China peninsula

**DOI:** 10.3897/zookeys.939.31764

**Published:** 2020-06-09

**Authors:** Guogang Li, Ye Htet Lwin, Bin Yang, Tao Qin, Phouthong Phothisath, Kyaw-Win Maung, Rui-Chang Quan, Song Li

**Affiliations:** 1 Southeast Asia Biodiversity Research Institute, Chinese Academy of Sciences & Center for Integrative Conservation, Xishuangbanna Tropical Botanical Garden, Chinese Academy of Sciences, Mengla, Yunnan, 666303, China; 2 Kunming Natural History Museum of Zoology, Kunming Institute of Zoology, Chinese Academy of Sciences, 32 Jiaochang Donglu, Kunming, Yunnan 650223, China; 3 Biotechnology and Ecology Institute, Ministry of Science and Technology of Laos, P. O. Box 2279, Vientiane Capital, Lao People's Democratic Republic; 4 Forest Research Institute, Forest Department, Ministry of Environmental Conservation and Forestry, Yezin, Nay Pyi Taw, 05282, Myanmar

**Keywords:** *
Biswamoyopterus
*, flying squirrel, Indo-China peninsula, taxonomic revision.

## Abstract

The flying squirrel genus *Biswamoyopterus* (Rodentia: Sciuridae: Pteromyini) was once considered to contain three species, *Biswamoyopterus biswasi* from northeastern India, *B. laoensis* from central Laos and *B. gaoligongensis* from southwest China, all identified from morphological characteristics of one or two specimens. However, based on similar morphological characteristics of two samples of the genus *Biswamoyopterus* collected recently from northern Laos and northern Myanmar, and the small genetic distances on mitochondrial DNA and nuclear DNA between them, the results strongly support these two samples as representatives of the same species. The phylogenetic analyses strongly support *Biswamoyopterus* as an independent genus of Pteromyini, as a sister group to *Aeromys*. *Biswamoyopterus biswasi* is distributed in the northern Indo-China peninsula, where it is exposed to a series of threats, such as intense hunting activity, illegal trade, and rapid habitat loss; this should warrant its classification as critically endangered according to the International Union for Conservation of Nature (IUCN) Red List criteria. Here, the molecular data for genus *Biswamoyopterus* and two new specimen records from northern Laos and northern Myanmar are presented.

## Introduction

Flying squirrels (Mammalia: Rodentia: Sciuridae: Pteromyini), occurring in northern coniferous forests to the tropical lowlands of North America and Eurasia, are great masters of gliding locomotion using well-developed membrane structures (Thorington et al. 2002). Pteromyini comprises 15 monophyletic genera nested within Sciuridae (Mercer and Roth 2003; Wilson and Reader 2005), with high external morphological diversification between genera. It is useful to understand the taxonomic theories behind these genera, based on skull characteristics and external morphology (Ellerman 1940; Ellerman and Morrison-Scott 1950; Corbet and Hill 1992; Nowak 1999; Thorington et al. 2002; Wilson and Reader 2005) (Table 1).

**Table 1.** Taxonomic hypotheses of various authors regarding Pteromyidae/Pteromyini.

Ellerman (1940)	Ellerman and Morrison-Scott (1950)	Corbet and Hill (1992)	Nowak (1999)	Thorington et al. (2002)
	* Aeretes *	* Aeretes *	* Aeretes *	* Aeretes *
* Aeromys *		* Aeromys *	* Aeromys *	* Aeromys *
* Belomys *	* Belomys *		* Belomys *	* Belomys *
		* Biswamoyopterus *	* Biswamoyopterus *	* Biswamoyopterus *
* Eoglaucomys *			* Eoglaucomys *	* Eoglaucomys *
* Eupetaurus *	* Eupetaurus *	* Eupetaurus *	* Eupetaurus *	* Eupetaurus *
* Glaucomys *			* Glaucomys *	* Glaucomys *
* Hylopetes *	* Hylopetes *	* Hylopetes *	* Hylopetes *	* Hylopetes *
* Iomys *		* Iomys *	* Iomys *	* Iomys *
* Petaurillus *		* Petaurillus *	* Petaurillus *	* Petaurillus *
* Petaurista *	* Petaurista *	* Petaurista *	* Petaurista *	* Petaurista *
* Petinomys *	* Petinomys *	* Petinomys *	* Petinomys *	* Petinomys *
* Pteromys *	* Pteromys *		* Pteromys *	* Pteromys *
* Pteromyscus *		* Pteromyscus *	* Pteromyscus *	* Pteromyscus *
* Trogopterus *	* Trogopterus *	* Trogopterus *	* Trogopterus *	* Trogopterus *

**Figure 1.**
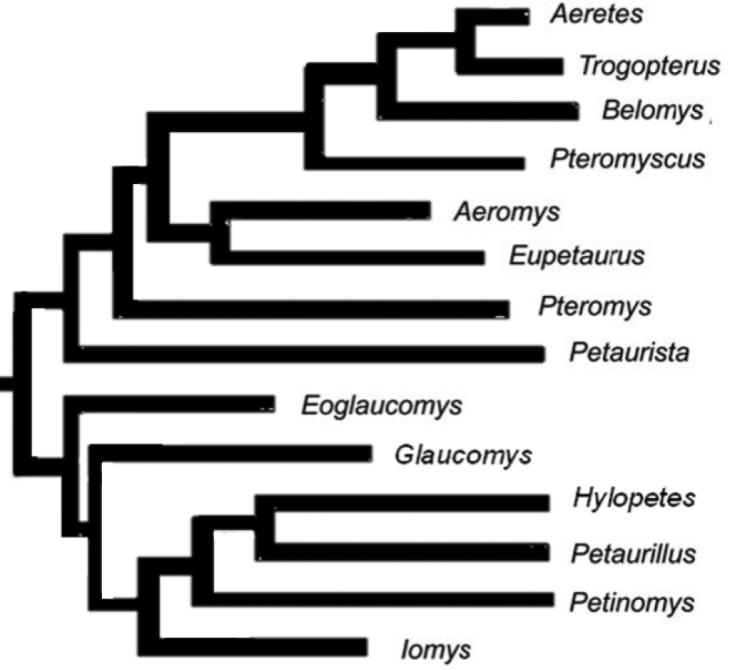
Phylogeny of Pteromyini genera. Cited from Mercer and Roth (2003).

**Figure 2.**
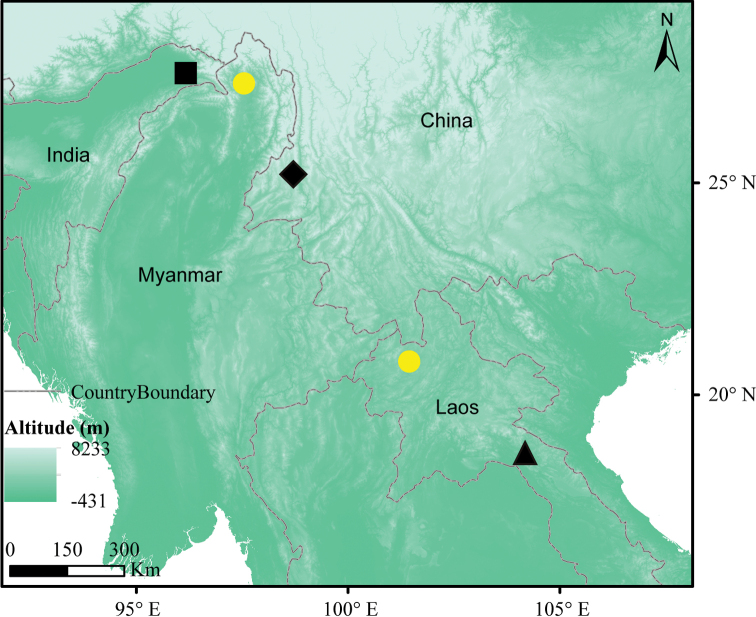
Localities of *Biswamoyopterus* specimens. The black square represents *B. biswasi*, ZSI 20705 (Saha, 1981); the black triangle represents *B. laoensis*, NUoL FES. MM.12.163 (Sanamxay et al. 2013); the black diamond represents *B. gaoligongensis*, KIZ 034924 and KIZ 035622 (Li et al. 2019); the yellow circles represent *Biswamoyopterus* sp., M644 and L35, collected in this study.

Many studies on the molecular phylogeny of Pteromyini genera have been performed since 2000 (Oshida 2000a, b, 2001, 2004; Mercer and Roth 2003; Yu et al. 2004, 2006; Lu et al. 2012); however, most of them were carried out with one or a few genera, and even the analyses by Mercer and Roth (2003), which examined 14 of the 15 genera, excluded the genus *Biswamoyopterus* (Figure 1). The genus *Biswamoyopterus* was described by Saha in 1981. Identified on respective morphological characteristics of one or two specimens, it comprises three species, *Biswamoyopterus biswasi* Saha, 1981 (specimen ZSI 20705) found in northeastern India, *B. laoensis*Sanamxay et al., 2013 (specimen NUoL FES. MM.12.163), found in central Laos, and *B. gaoligongensis*Li et al., 2019 (specimens ZSI 20705 & KIZ 034924), found in southwest China (Figure 2, see Saha 1981; Sanamxay et al. 2013; Li et al. 2019). No molecular data have been obtained about this genus so far.

Since 2014, the Southeast Asia Biodiversity Research Institute, Chinese Academy of Sciences (CAS-SEABRI), has conducted several biodiversity expeditions to the northern Indo-China peninsula (Li and Quan 2017; Li et al. 2017). This region is considered a globally important biodiversity hotspot for flora and fauna (Tordoff et al. 2005), from where many species of mammals have been discovered or rediscovered since the 1990s (Amato et al. 1999; Geissmann et al. 2011; Sanamxay et al. 2013; Fan et al. 2017). In this work, using combined mitochondrial DNA and nuclear DNA loci, and morphological examination, we aim to revise the taxonomic status of the genus *Biswamoyopterus* and assess its phylogenetic position among flying squirrels.

## Materials and methods

### Ethics statement

All samples used in this study were obtained by the CAS-SEABRI expeditions on the northern Indo-China peninsula, with export permits (no. L/2020-0001/MA-0004/MA) issued by Biotechnology and Ecology Institute, Ministry of Science and Technology of Lao PDR, and permission (1567/XTBG/2017) issued by the Forest Research Institute, Forest Department, Ministry of Environmental Conservation and Forestry of Myanmar.

### Materials

Twelve flying squirrel samples (two of *Biswamoyopterus* and ten of *Petaurista*) were collected from northern Myanmar and northern Laos during the expedition of 2014–2018 (see Suppl. material 1: Table S1). The samples M644 and L35 were recognized as belonging to the genus *Biswamoyopterus*. Specimen M644 (whole body) was collected from a local market in Putao county (27°20'31.20"N, 97°24'3.60"E; 446 m asl), Kachin State, Myanmar (Figure 2), on 24 November 2017, and has been deposited in CAS-SEABRI Myanmar Lab, Nay Pyi Taw, Myanmar. Specimen L35 was photographed (Suppl. material 2: Figure S1) in a local market in Louang Namtha, northern Laos (Figure 2) on 27 March 2018, and only some tissue was collected for molecular data analysis. All sequences have been deposited in GenBank (accession numbers MK105519–MK105539); detailed sequence information has been listed in Suppl. material 1: Table S1.

### Morphological methods

**Figure 3.**
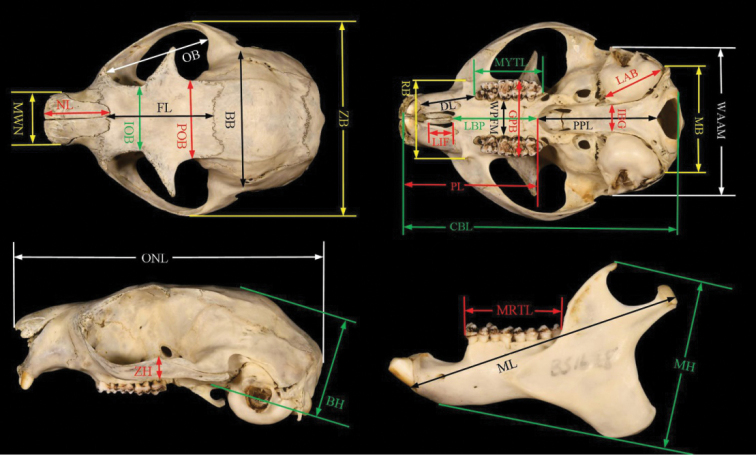
Illustration of the 28 cranial variables measured in this study was derived from Li et al. (2019).

According to the taxonomic assignments of Wilson and Reader (2005), pelage and skull characteristics can be discriminated using traditional methods and compared with those of other genera using specimens (Appendix I) retained in the Kunming Natural History Museum of Zoology, Kunming Institute of Zoology, Chinese Academy of Sciences (**KIZ**) (Kunming, China); the Institute of Zoology, Chinese Academy of Sciences (**IOZ**) (Beijing, China); and the Guangdong Entomological Institute (**GDEI**) (Guangzhou, China); or using documented literature (Gunther 1873; Robinson and Kloss 1915; Ellerman 1940; Corbet and Hill 1992; Nowak 1999). Following the results of Li et al. (2019), 28 cranial variables were measured with a digital caliper to the nearest 0.01 mm and these are presented in Table 2 and Figure 3:

**Table 2.** Comparison of five specimens of genus *Biswamoyopterus*. M644 was measured (millimeters) in this study, others were derived from Li et al. (2019).

Specimen	*B. biswasi*	*B. gaoligongensis*	*B. gaoligongensis*	*B. laoensis*	*Biswamoyopterus* sp. M644
Sex	male	male	unknown	female	unknown
Locality	Northeastern India	Southwestern China	Southwestern China	Central Laos	Northern Myanmar
Head and body length	405	440	–	455	540
Tail length	605	520	–	620	605
Hind feet length	78	75	–	74.5	71
Ear length	46	47	46	52	43
ONL	72.4	69.75	71.11	74.39	74.22
CBL	70.1	66.37	67.73	70.99	69.88
MB	–	30.72	33.5	30.79	27.15
ZB	47.5	48.41	48.3	47.72	47.09
ZH	–	4.61	4.58	4.86	5.03
BB	–	33.86	34.46	32.84	33.68
BH	–	22.9	24.15	22.55	22.37
RB	–	19.61	19.62	17.04	19.66
NL	20.9	19.35	20.7	22.57	21.83
MWN	–	13.15	12.51	13.37	13.23
IOB	19	15.75	16.38	14.06	14.29
POB	–	18.87	20.55	17.19	16.87
LIF	6.4	5.65	5.86	5.85	6.21
LBP	–	20.08	22.01	23.83	22.37
PPL	–	28.72	29.68	28.77	29.96
LAB	15.5	14.68	14.57	17.33	15.03
WAAM	–	35.88	36.76	35.96	36.96
IBG	–	6.52	6.76	5.01	6.41
MYTL	15.5	15.92	16.23	16.33	16.53
GPB	–	18.26	18.61	19.37	19.98
WPFM	–	8.58	8.03	8.05	8.34
MRTL	–	15.24	15.41	15.33	15.75
ML	–	44.44	46.53	45.36	44.67
MH	–	27.1	27.37	29.78	29.66
PL	34.7	32.6	32.87	–	35.08
DL	15.7	13.7	15.03	–	15.30
OB	24.6	26.17	26.5	–	28.42
FL	28.6	27.66	30.63	–	30.27

**BB** Breadth of braincase,

**BH** Braincase height,

**CBL** Condylobasal length,

**DL** Diastema length,

**FL** Frontal length,

**GPB** Greatest palatal breadth,

**IBG** Inter bullae gap,

**IOB** Interorbital breadth,

**LAB** Length of auditory bulla,

**LBP** Length of bony palate,

**LIF** Length of the incisive foramina,

**MB** Mastoid breadth,

**MH** Mandible height,

**ML** Mandible length,

**MRTL** Mandibular tooth row length,

**MWN** Maximum width of nasals,

**MYTL** Maxillary tooth row length,

**NL** Nasal length,

**OB** Orbit breadth,

**ONL** Occipitonasal length,

**PL** Palate length,

**POB** Postorbital breadth,

**PPL** Postpalatal length,

**RB** Rostrum breadth,

**WAAM** Width of auditory bullae across the external auditory meati,

**WPFM** Width of the bony palate at the first upper molar,

**ZB** Zygomatic breadth,

**ZH** Zygomatic height,

**P** Premolars,

**M** Molars.

Superscript (P^X^, M^X^) upper premolars and upper molars, and Subscript (P^X^, M^X^) lower premolars and lower molars.

**Figure 4.**
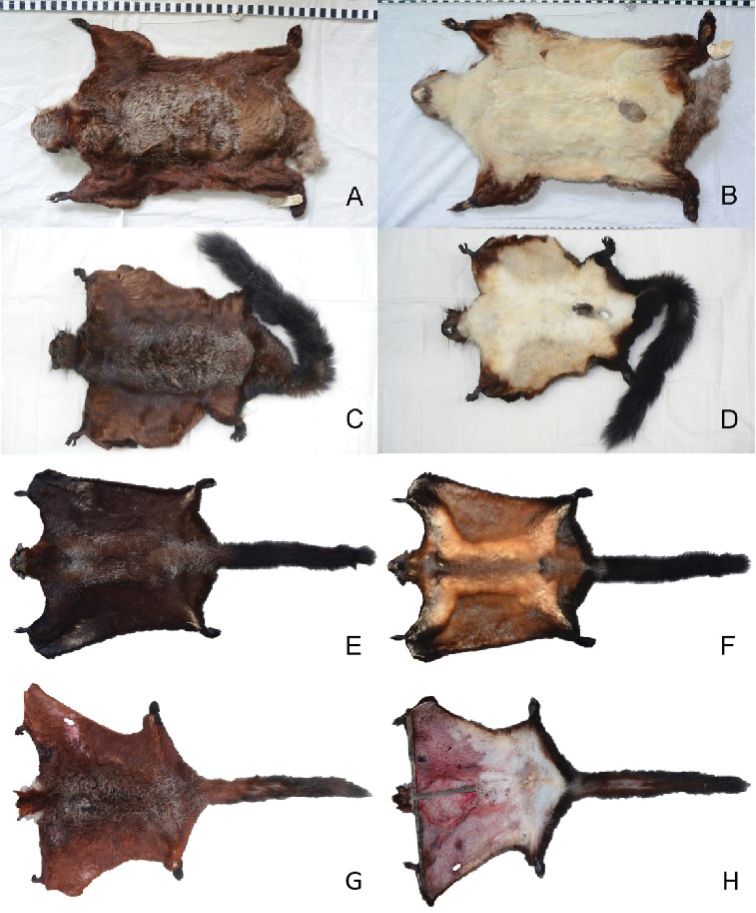
Comparison of skins of all known *Biswamoyopterus* specimens **A, B***B. biswasi*, ZSI 20705 **C, D***B. gaoligongensis*, KIZ 034924 **A–D** were derived from Li et al. (2019)**E, F***B. laoensis*, NUoLFES.MM.12.163, from Sanamxay et al. (2013)**G, H***Biswamoyopterus* sp. M644 from this study.

**Figure 5.**
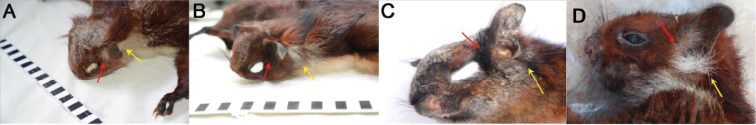
Comparison of ear tufts of all known *Biswamoyopterus* specimens. The red arrow indicates the anterior tufts, and the yellow arrow indicates the posterior tufts **A***B. biswasi*, ZSI 20705 **B***B. gaoligongensis*, KIZ 034924 **A, B** were derived from Li et al. (2019)**C***B. laoensis* NUoL FES.MM.12.163 from Sanamxay et al. (2013)**D***Biswamoyopterus* sp. M644 from this study.

**Figure 6.**
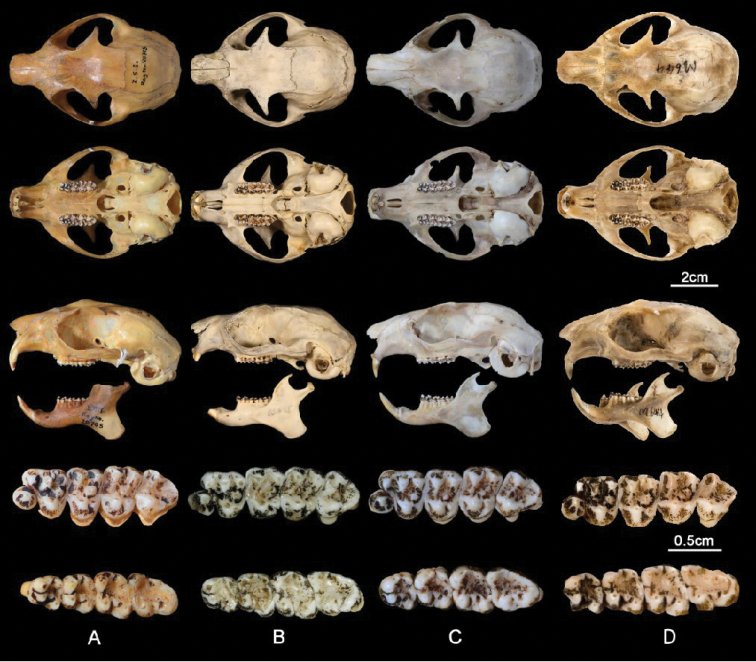
The skulls (first three rows), left maxillary (the fourth rows) and left mandibular teeth (the last rows) of all known *Biswamoyopterus* specimens **A***B. biswasi*, ZSI 20705 **B***B. gaoligongensis*, KIZ 034924 **A, B** were derived from Li et al. (2019)**C***B. laoensis*, NUoL FES.MM.12.163, from Sanamxay et al. (2013)**D***Biswamoyopterus* sp. M644 from this study.

**Figure 7.**
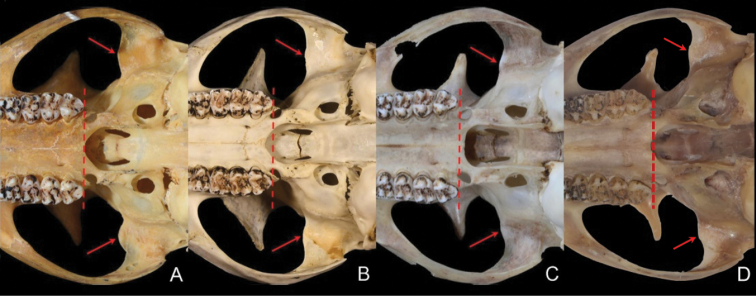
The posterior margin of the palatal bones relative to the posterior margin of M3 (dotted line) and shape of the preglenoid process (arrow) of all known *Biswamoyopterus* specimens **A***B. biswasi*, ZSI 20705 **B***B. gaoligongensis*, KIZ 034924) **A, B** were derived from Li et al. (2019)**C***B. laoensis*, NUoL FES.MM.12.163, from Sanamxay et al. (2013)**D***Biswamoyopterus* sp. M644 from this study.

In addition, measurements of the head and body length, tail length, hind foot length, and ear length were taken and compared with the original measurements labeled on the skins by the collectors. The skull measurements of M644 are listed in Table 2. Figures 4–7 display, respectively, the pelage and skull characteristics of M644 compared with all known *Biswamoyopterus* specimens, according to Saha (1981), Sanamxay et al. (2013), and Li et al. (2019).

### Molecular data and analyses

Total genomic DNA was extracted from tissue using a DNeasy Blood & Tissue kit (Qiagen, Shanghai, China). PCR mixtures contained approximately 100 ng of template DNA, 1 μL (10 pmol) of each primer, 5 μL of 10× reaction buffer, 2 μL of dNTPs (2.5 mM of each), and 2.0 U of Taq DNA polymerase, in a total volume of 50 μL. Reactions were carried out in a Veriti Thermal Cycler (Applied Biosystems, Carlsbad, CA, USA) and always included a negative control. Segments of the nuclear genes encoding the inter photoreceptor retinoid-binding protein (IRBP) and mitochondrial 12S and 16S ribosomal DNA of flying squirrels were amplified using PCR with universal primers described previously (Mercer and Roth 2003; Guha et al. 2007). Fragments were visualized using electrophoresis in 1% agarose gel, and PCR products were sequenced from both ends using an ABI PRISM 3700 sequencing system, using the same primers as for PCR (Beijing Tianyi Huiyuan Bioscience and Technology Incorporation, Beijing, China).

DNA sequences were edited using the DNASTAR 5.0 (DNASTAR Inc.) program and aligned using the CLUSTALW algorithm in MEGA 6.06, with default parameters (Larkin et al. 2007; Tamura et al. 2013). Identical haplotypes were collapsed using DNASP 5.1 (Librado and Rozas 2009), and the base composition of mitogenomic sequences was analyzed using MEGA 6.06 (Tamura et al. 2013).

Phylogenies using the combined mitochondrial and nuclear DNA data from our collection and GenBank were reconstructed using maximum likelihood in RaxML version 8 (Stamatakis 2014) and Bayesian Inference (BI) in MRBAYES 3.2.6 (Ronquist et al. 2012), while the most appropriate nucleotide substitution models were selected using the Akaike Information Criterion in jMODELTEST 2.1.4 (Darriba et al. 2012). The significance of the hypothesized lineages from maximum-likelihood analyses was tested using Bootstrap analysis with 200 replicates with default settings. Markov Chain Monte Carlo (MCMC) analysis approximated posterior distributions with one cold and three heated chains, and samples of the trees and parameters were drawn every 100 steps from a total of one million MCMC generations; three additional runs were conducted beginning with random trees. The 50% majority rule consensus of the post-burn (using a burn-in of 25%) for all generations was computed for the four runs. Trees were visualized using FIGTREE 1.4 (Rambaut and Drummond 2012). Sequences representing *Tamiasciurus hudsonicus* and *Ratufa bicolor* were obtained from GenBank and used as outgroups to root the tree (Mercer and Roth 2003). Average genetic divergence was calculated between and within the studied flying squirrel species in MEGA 6.06 (Tamura et al. 2013).

## Results

### Morphological description of Biswamoyopterus

Taxon classificationAnimaliaRodentia Sciuridae

sp. M644

4AE17787-B7AB-5427-9F41-4D91D77CDCA0

Figures 4G, H , 5D , 6D , 7D

#### Remarks.

Morphometrical data are presented in Table 2. As a whole, the dorsal pelage is reddish brown, with dense whitish hairs on the shoulders and hips, the ventral pelage is whitish, with yellowish brown on the edge of the membrane, the anus area is dull yellowish, but the base of the tail is brown-grey. The ears are black with few hairs, but with tufts of long, whitish hairs at the base. The feet backs are covered with black hairs, and the tail is cylindrical and reddish brown in its anterior part but gradually tending to blackish brown in its distal part. The skull is large with a GLS of 74.77 mm and a ZOB of 47.09 mm. The bullae are enlarged and each of them includes numerous septa (> 10) in a complex honeycomb pattern. The anterior edge of the nasals is slightly arc-shaped and extends slightly beyond the surface of the incisors. The surfaces of the upper and lower incisors are dull yellowish, without any orange. In the cheek teeth, P^3^ is relatively enlarged and cone-shaped. The length of P^4^ slightly exceeds each of the molars; P^4^ has three well-developed cusps on the labial side and one large cusp on the lingual side. Both M^1^ and M^2^ have two well-developed cusps on the labial side and one large cusp and one smaller cusp on the lingual side, and there is a smaller cusp on the posterior transverse ridge of P^4^, M^1^, and M^2^. M^3^ is smaller than P^4^, M^1^, and M^2^, and its later crown surface becomes a “U” shape, with a slight depression in its center.

The upper surface of the head is deep reddish brown, the muzzle is brown, the rim of the eyes is brown, the cheeks are reddish brown with occasional whitish hairs on their lower parts, the ears are black with few hairs but tufts with long, whitish hairs at the base, the back of the neck is reddish brown, and the throat and chin show whitish grey extending to both sides of the neck.

The back is mainly reddish brown, but is scattered with many white tips, especially on the shoulders and hips; individual hairs are variable in color but usually comprise the following components: whitish at the tip, reddish brown in the mid-part, and whitish grey at the base. The anterior margin of the forearms is black-brown. The chest is yellowish grey, the center of the abdomen is yellowish white, and the anus area is dull yellowish. The upper part of the membrane is reddish brown and the underpart whitish, extending to yellowish brown on the edge. The tail is cylindrical, reddish brown anteriorly, but gradually darkening towards the tip, so its posterior part is blackish brown, and the underpart area of the tail base is brown-grey. The fore and hind feet are covered with black hairs; however, the hind feet have denser hair than the fore feet, and both have dark hairless pads.

The skull is large, the frontal part is significantly depressed, the rostrum is short and wide, the anterior edge of the nasals is slightly beyond the surface of the incisors with a slight arc-shape, the incisive foramen is developed, the palatine posterior edge has an arc-shaped depressed deformation, the pterygoid is strong and the pterygoid fossa wider, the bulla is developed with numerous septa (> 10) in a complex honeycomb pattern, the orbital regions are large and there is an incision on the edge of each orbit, the postorbital process is strong and curves down a little, the zygomatic plate is slant, the zygomatic arch is stronger with lower connection to the squamosal, the mastoid process is comparatively smaller, but the occipital condyle is strong.

The mandible is strong, with the coronoid process developed, and the condylar process has a developed articular surface; the angular process is developed and curved towards the inside at its bottom. The upper incisors are strong and positioned vertically downwards; their outer surfaces are yellowish, without any orange. P^3^ is cone-shaped and on the inside of the front of P^4^; overall, the crown surface of P^4^ appears as a triangle with three well-developed cusps on the labial side and one large cusp on the lingual side, and its labial side length is slightly longer than those of M^1^, M^2^, and M^3^. M^1^ and M^2^ are approximately equal in size; both have two well-developed cusps on the labial side, and one large cusp and one smaller cusp on the lingual side. There is a smaller cusp on the posterior transverse ridge of P^4^, M^1^, and M^2^. Compared with P^4^, M^1^, and M^2^, M^3^ is the smallest; its lingual side cusp is larger than the cusp on the labial side, and its later crown surface becomes a U-shape, with a small depression in its center.

The outer surface of the lower incisors is yellowish, the same as for the upper incisors; however, the inside part of the inner surface sinks deeply, making the outside margin sharp. From P_4_ to M_3_, the teeth enlarge gradually, and there are two labial and lingual cusps on each of them (the later lingual cusp of M_3_ becomes a ridge); there is also a smaller cusp between, and slightly internal to, the two labial cusps on each of them. Different levels of depression occur in the centers of the crown surfaces of P_4_, M_1_, M_2_, and M_3_, with the largest in M_3_.

### Morphological description of Biswamoyopterus

Taxon classificationAnimaliaRodentia Sciuridae

sp. L35

45C70733-0599-57C7-BF11-7B2D5090D7BD

Table 3 , Suppl. material 2: Figure S1

#### Remarks.

The sample L35 from northern Laos shares the same pelage color of the tuft hair at the base of the ear and side of the neck (Figure 5, Suppl. material 2: Figure S1) with the *Biswamoyopterus laoensis* specimen (NUoL FES. MM.12.163) from central Laos. However, specimen M644 from northern Myanmar shares some key characters that have been used to distinguish the three known species from each other (Figures 4–7, Table 3): its large body size and long muzzle are similar to *B. laoensis* (NUoL FES. MM.12.163) from central Laos; the coloration of venter, tail, and ear tufts could pertain to either *B. biswasi* (specimen ZSI 20705) from northeastern India or *B. gaoligongensis* (specimen KIZ 034924) from southwestern China, which are very similar.

**Table 3.** Comparison of five specimens of genus *Biswamoyopterus*. M644 and L35 were described in this study, others were derived from Li et al. (2019).

Specimen	*B. biswasi*, ZSI 20705, ♂	*B. gaoligongensis*, KIZ 034924, ♂	*B. laoensis*, NUoL FES. MM.12.163, ♀	*Biswamoyopterus* sp. M644	*Biswamoyopterus* sp. L35
Locality	Northeastern India	Southwestern China	Central Laos	Northern Myanmar	Northern Laos
Size	Relatively small	Relatively small	Large	Large	Large
Dorsal coloration	Morocco-red speckled with white	Reddish brown speckled with white	Dark reddish brown speckled with whitish grey	Reddish brown speckled with whitish	Dark reddish brown speckled with whitish grey
Ventral Coloration	Light colored		Pale orange and marked with numerous, black, discontinuous lines	White	
	White	Yellowish-white			
Coloration of tail beyond the uropatagium	Partly colored tail with a dark tip		Black	Reddish brown with a brown-grey tip	
	Pale smoky grey with a dark tip	Black			
Ear tufts	Bicolored or white		Black	White	White
	White	The anterior tufts are black, and the posterior tufts are basally white and terminal black			
NL	Short		Long	Long	–
	Short	Shorter			
Outer margin of the nasal bone, orbital margin of the frontal bone, and post-orbital margin of the frontal bone vs. midline of the skull	Inclined	Almost	More	Inclined	–
Postorbital processes	Large	Large	Relatively small	Large	–
Preglenoid process	Forward protruding	Almost flat	Almost flat	Almost flat	–
Sutures of frontal and squamosal bone	Almost flat	Bulge	Almost flat	Almost flat	–
Auditory bulla	Smaller		Large	Relatively small	–
	Relatively small	Relatively small			
Posterior margin of the palatal bones	Concave forward		Flat	Concave forward	–
	The central point just meets the posterior margin of M^3^	The central point lies in front of the posterior margin of M^3^	The central point lies behind the posterior margin of M^3^	The central point lies just a little in front of the posterior margin of M^3^	
M^1^ and M^2^	Feeble metacone and hypocone, outline of M^1^ and M^2^ is sub-triangular	Most developed metacone and hypocone, outline of M^1^ and M^2^ is sub-square	Second developed metacone and hypocone, outline of M^1^ and M^2^ is sub-rectangle	Second developed metacone and hypocone, outline of M^1^ and M^2^ is sub-rectangle	–
M_1_ and M_2_	Second developed hypoconid	Most developed hypoconid	Feeble hypoconid	Feeble hypoconid	–

#### Phylogeny and genetic divergence.

**Figure 8.**
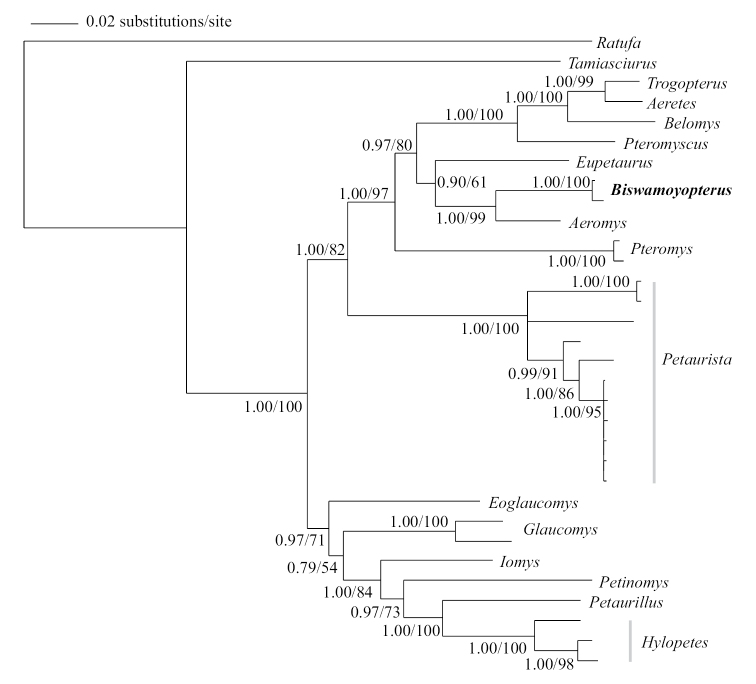
Bayesian Inference and Maximum Likelihood inference tree (GTR+G+I model) of flying squirrels based on combined mitochondrial and nuclear DNA data. Numbers on branches indicate posterior probability in BI and bootstrap support from ML.

Maximum Likelihood and Bayesian Inference analyses of the combined sequences of nuclear gene IRBP (1070 bp), mitochondrial 12S (823 bp), and 16S (535 bp) ribosomal DNA recovered similar tree topologies. The results showed that *Eupetaurus*, *Aeromys*, and *Biswamoyopterus* (sample M644 from Putao, northern Myanmar, and L35 from Louang Namtha, northern Laos) as a reciprocally monophyletic clade (Figure 8). Within this clade, *Aeromys* and *Biswamoyopterus* form sister groups with strong support (Figure 8).

For the nuclear gene IRBP, the range of original intergeneric (14 genera excluding the genus *Biswamoyopterus*) variation was 0.51–5.47% (Table 4). The genetic distances between *Biswamoyopterus* and other genera ranged from 1.57 to 5.27% (Table 4), which is greater than many intergeneric variations, such as 0.51% for *Aeretes* and *Trogopterus*, 1.46% for *Belomys* and *Pteromyscus*, and 1.46% for *Pteromyscus* and *Trogopterus*. In the genus *Biswamoyopterus*, the genetic distance between M644 and L35 was 0.09%, which is smaller than the range of other interspecific variations (0.51–5.45%), close to 0.13% for intraspecific variations of *Pteromyscus pulverulentus*.

**Table 4.** Average genetic distances (%) for nuclear IRBP-encoding sequences between the groups of studied flying squirrel species; intraspecific variations of genetic distances are also provided for each species.

	mel	tep	pea	cin	fim	vol	pha	hor	fus	phi	kin	ele	pul	set	ans	xan	Bis
*Aeretes melanopterus* (mel)																	
*Aeromy tephromelas* (tep)	2.86																
*Belomys pearsonii* (pea)	1.37	2.86															
*Eupetaurus cinereus* (cin)	2.68	2.16	3.03														
*Eoglaucomys fimbriatus* (fim)	4.09	4.01	4.19	3.74													
*Glaucomys volans* (vol)	5.18	4.90	5.00	4.64	3.47												
*Hylopetes phayrei* (pha)	3.94	4.20	3.76	4.20	3.13	3.66											
*Iomys horsfieldi* (hor)	3.92	4.01	3.76	3.84	2.69	2.95	2.60										
*Petaurista alborufus* (fus)	4.36	4.27	4.82	4.27	4.27	4.73	4.19	4.28									
*Petaurista philippensis* (phi)	3.77	4.08	4.39	3.77	3.67	4.49	4.09	3.67	1.06								
*Petaurillus kinlochii* (kin)	4.18	4.19	4.19	3.91	1.89	3.38	2.42	2.16	4.46	3.97							
*Petaurista elegans* (ele)	4.75	4.95	5.38	4.75	4.44	4.44	4.76	4.24	2.18	1.46	4.54						
*Pteromyscus pulverulentus* (pul)	1.28	2.77	1.46	2.59	3.92	4.91	3.85	3.66	4.74	3.88	3.91	4.85					
*Petinomys setosus* (set)	4.28	4.65	4.38	4.10	3.03	3.92	2.87	2.69	4.82	4.08	2.77	4.86	4.10				
*Pteromys volans* (ans)	4.00	3.91	3.83	3.91	4.18	5.08	4.29	4.47	4.99	4.90	4.55	5.47	3.75	4.56			
*Trogopterus xanthipes* (xan)	0.51	3.21	1.54	3.03	4.38	5.37	4.11	4.11	4.65	3.98	4.37	4.86	1.46	4.56	4.01		
*Biswamoyopterus* sp. (bis)	3.06	1.57	3.26	2.52	4.14	5.04	4.35	3.69	4.74	4.29	4.18	5.27	2.77	4.64	4.14	3.36	
Intraspecific variations	n/c	n/c	n/c	n/c	n/c	n/c	n/c	n/c	n/c	0.13	n/c	n/c	n/c	n/c	n/c	n/c	0.09

For the mitochondrial 16S ribosomal DNA sequences, the range of original intergeneric variation was 2.9–14.6% (Table 5). The genetic distances between *Biswamoyopterus* and other genera ranged from 5.2 to 12.8% (Table 5), which is greater than some intergeneric variations, such as 2.9% for *Aeretes* and *Trogopterus*. In the genus *Biswamoyopterus*, the genetic distance between M644 and L35 was 0.6%, which is really much smaller than the range of other interspecific variations (2.9–14.6%), close to 0.4% for intraspecific variations of *Petaurista philippensis*, and the same as 0.6% of *Pteromys volans*.

**Table 5.** Average genetic distances (%) for 16S ribosomal DNA sequences between the groups of studied flying squirrel species; intraspecific variations of genetic distances are also provided for each species.

	mel	tep	pea	cin	fim	vol	pha	alb	hor	fus	hai	yun	phi	kin	ele	pul	set	ans	xan	Bis
*Aeretes melanopterus* (mel)																				
*Aeromy tephromelas* (tep)	9.5																			
*Belomys pearsonii* (pea)	6.5	11.6																		
*Eupetaurus cinereus* (cin)	10.4	8.2	11.5																	
*Eoglaucomys fimbriatus* (fim)	11.5	11.6	12.4	11.8																
*Glaucomys volans* (vol)	9.7	9.8	12.2	11.3	8.7															
*Hylopetes phayrei* (pha)	11.1	11.7	12.5	12.5	10.8	8.5														
*Hylopetes alboniger* (alb)	10.9	11.4	12.2	12.3	10.5	8.4	2.9													
*Iomys horsfieldi* (hor)	9.8	9.7	10.3	11.0	9.2	7.6	7.3	7.5												
*Petaurista alborufus* (fus)	11.3	10.9	12.4	12.1	13.2	11.6	10.6	11.1	10.5											
*Petaurista hainana* (hai)	12.2	11.8	12.4	11.3	13.4	11.3	11.7	11.3	10.1	7.6										
*Petaurista yunanensis* (yun)	11.7	11.6	12.9	11.1	12.7	11.0	10.3	10.2	9.8	6.1	2.3									
*Petaurista philippensis* (phi)	12.4	11.8	13.9	12.4	12.5	11.2	11.0	11.1	10.3	7.6	2.7	1.9								
*Petaurillus kinlochii* (kin)	9.4	10.6	11.0	11.5	9.0	7.4	7.1	8.0	5.6	11.2	9.7	8.8	9.9							
*Petaurista elegans* (ele)	11.9	13.0	12.6	12.5	13.4	13.0	10.4	10.0	10.9	7.9	6.5	6.6	6.8	10.5						
*Pteromyscus pulverulentus* (pul)	8.0	11.1	7.8	11.5	13.6	12.3	13.8	13.6	11.4	12.4	13.3	12.6	13.6	10.8	12.7					
*Petinomys setosus* (set)	11.7	11.2	12.9	12.7	8.4	10.6	8.9	8.6	10.1	12.9	13.3	12.1	12.6	9.0	11.7	14.6				
*Pteromys volans* (ans)	10.2	10.5	10.9	10.6	10.5	10.9	12.2	11.7	10.1	12.4	10.9	10.7	11.2	8.3	12.2	11.6	11.4			
*Trogopterus xanthipes* (xan)	2.9	10.4	6.1	10.4	12.2	10.7	10.9	10.9	9.6	11.3	12.2	11.7	12.4	9.2	11.4	8.0	12.1	10.6		
*Biswamoyopterus* sp. (bis)	8.9	5.2	8.9	8.0	8.9	9.3	9.9	9.6	8.8	11.0	12.3	11.1	11.1	9.5	12.8	10.9	11.2	10.3	8.5	
Intraspecific variations	n/c	n/c	n/c	n/c	n/c	3.3	5.1	n/c	n/c	n/c	n/c	n/c	0.4	n/c	0.4	n/c	n/c	0.6	n/c	0.6

## Discussion

According to morphological comparisons of our samples and those from previous studies (Saha 1981; Sanamxay et al. 2013; Li et al. 2019), *Biswamoyopterus* specimens L35 from northern Laos and M644 from northern Myanmar are confirmed as representing the genus *Biswamoyopterus*. However, *Biswamoyopterus* sp. M644 shares many key characters with all three known *Biswamoyopterus* species. Since each *Biswamoyopterus* species has been described on the basis of only one or two samples, it is possible that the observed morphological differences are the result of intraspecific variation. If so, it is plausible that all known *Biswamoyopterus* specimens might in fact be conspecific.

It was further implied by the molecular evidence that samples L35 and M644 belonged to the same species, with the smallest nuclear and mitochondrial DNA genetic distance among interspecific variations for any of the studied flying squirrel species (Tables 4, 5). Sanamxay et al. (2013) distinguished *B. laoensis* from *B. biswasi* mainly by 1) the large distance of 1250 km between the localities of the two species and 2) the different pelage colors present mostly on the ventral side: “white but washed with a faint orange-rufous” in *B. biswasi* versus “essentially orange” in *B. laoensis*. These factors were also true for samples L35 and M644, being separated by a long distance of more than 1000 km and different ventral pelage colors. Eliécer and Guilherme (2018) performed a study on species delimitation based on diagnosis and monophyly. The current molecular results and the morphological variability observed between *Biswamoyopterus* specimens M644 and L35 indicate that further studies should be performed to shed light on the relationships among *B. biswasi*, *B. laoensis*, and *B. gaoligongensis*.

The molecular phylogenetic analysis strongly supported *Biswamoyopterus* as an independent genus within Pteromyini, acting as a sister group to *Aeromys* (Figure 8). For nuclear and mitochondrial DNA sequences, the genetic distances between *Biswamoyopterus* and other genera are greater than many of the intergeneric variations (Tables 4, 5). Both nuclear and mitochondrial analyses suggested that *Biswamoyopterus* is a separate flying squirrel genus distinct from every validly described genus. We note that DNA sequences for genus *Aeretes* cited in the literature may be based on mistaken institutional identifications, as reported recently by Roth and Mercer (2015). Therefore, additional molecular evidence is needed to determine the phylogenetic relationships among these flying squirrels more clearly in the future.

During the expedition of 2014–2018, only two samples of *Biswamoyopterus* were found. We therefore propose that *Biswamoyopterus* should be classified as critically endangered on the IUCN Red List, due to a series of threats on the Indo-China peninsula that include intense hunting, illegal trade, and rapid habitat loss (Rao et al. 2010; Geissmann et al. 2011). In order to understand the population status, range, and other biological features of *Biswamoyopterus*, further studies including biodiversity expeditions covering the whole Indo-China peninsula should be performed. With respect to biogeography, members of the genus *Biswamoyopterus* inhabit the northern Indo-China peninsula, which belongs to one of the global biodiversity hotspot regions (Myers et al. 2000). The mechanisms responsible for their differentiation and how they have adapted to the environment are still unknown; therefore, more studies should be carried out to explore the differentiation, adaptation, and evolution of genus *Biswamoyopterus* and to make every effort to conserve them.

## Supplementary Material

XML Treatment for Biswamoyopterus

XML Treatment for Biswamoyopterus

## Appendix I

Specimens examined (IOZ, Institute of Zoology, Chinese Academy of Sciences; GDEI, Guangdong Entomological Institute; KIZ, Kunming Institute of Zoology, Chinese Academy of Sciences).

*Belomys*: KIZ 61004, 630743, 630799, 72226, 200362, 200363. *Petaurista*: IOZ 10457, 10458, 10460, 15041, 15042, 15043, 15044, 24009, 25849, 61-003. KIZ 73442, 73445, 73744, 73745, 73823, 830207, 90039, 90043, 90051, 90407. GDEI 0403, 0404, 0499, 0524, 0611, 0618, 0621, 0622, 0623, 0624, 0625, 0626. *Trogopterus*: KIZ 57048, 630784, 640575, 73377, 88637. *Aeretes*: KIZ 57052. *Eupetaurus*: KIZ 73372, 73373. *Pteromys*: KIZ 57053. *Hylopetes*: KIZ 73281, 74543, 74544, 74546, 76332, 76658.

## Supplementary materials

Supplementary material 1 Table S1. GenBank numbers of sequences that were analyzed in this study Data type molecular data https://binary.pensoft.net/file/419815This dataset is made available under the Open Database License (http://opendatacommons.org/licenses/odbl/1.0/). The Open Database License (ODbL) is a license agreement intended to allow users to freely share, modify, and use this Dataset while maintaining this same freedom for others, provided that the original source and author(s) are credited.Guogang Li, Ye Htet Lwin, Bin Yang, Tao Qin, Phouthong Phothisath, Kyaw-Win Maung, Rui-Chang Quan, Song Li

Supplementary material 2 Figure S1. Photograph of specimen L35 from northern Laos Data type multimedia https://binary.pensoft.net/file/419816This dataset is made available under the Open Database License (http://opendatacommons.org/licenses/odbl/1.0/). The Open Database License (ODbL) is a license agreement intended to allow users to freely share, modify, and use this Dataset while maintaining this same freedom for others, provided that the original source and author(s) are credited.Guogang Li, Ye Htet Lwin, Bin Yang, Tao Qin, Phouthong Phothisath, Kyaw-Win Maung, Rui-Chang Quan, Song Li
