# Supplementary material for: Taxonomic revision and phylogenetic position of the flying squirrel genus Biswamoyopterus (Mammalia, Rodentia, Sciuridae, Pteromyini) on the northern Indo-China peninsula
Source: Zookeys. 2020 Jun 9;939:65–85. doi: 10.3897/zookeys.939.31764 (PMC7341418; doi:10.3897/zookeys.939.31764)
Supplement: Supplementary material 1 — Table S1. GenBank numbers of sequences that were analyzed in this study [file zookeys-939-065-s001.docx]

**Table S1** Detailed information for specimens included in this study.

| Species name | Haplotype | GenBank Accession No. | | |
| --- | --- | --- | --- | --- |
|  |  | IRBP | 16S | 12S |
| Ingroup |  |  |  |  |
| *Biswamoyopterus* sp. M644 | M644^#^ | MK105534 | MK105519 | MK105526 |
| *Biswamoyopterus* sp. L35 | L35^#^ | MK105535 | MK105520 | MK105527 |
| *Petaurista philippensis* | L67^#^ | MK105536 | MK105521 | MK105528 |
| *Petaurista philippensis* | L84^#^ | MK105537 | MK105521 | MK105529 |
| *Petaurista philippensis* | L85^#^ | MK105536 | MK105521 | MK105528 |
| *Petaurista philippensis* | L147^#^ | MK105537 | MK105521 | MK105530 |
| *Petaurista philippensis* | L148^#^ | MK105537 | MK105522 | MK105531 |
| *Petaurista philippensis* | L151^#^ | MK105537 | MK105523 | MK105532 |
| *Petaurista philippensis* | L181^#^ | MK105538 | MK105521 | MK105533 |
| *Petaurista elegans* | L86^#^ | MK105539 | MK105524 |  |
| *Petaurista elegans* | L101^#^ | MK105539 | MK105525 |  |
| *Petaurista elegans* | L150^#^ | MK105539 | MK105524 |  |
| *Aeretes melanopterus* | Ame | AY227593 | AY227481 | AY227535 |
| *Trogopterus xanthipes* | Txa3 | AY227606 | AY227494 | AY227546 |
| *Belomys pearsonii* | Bpe | AY227595 | AY227483 | AY227537 |
| *Pteromyscus pulverulentus* | Ppu3 | AY227603 | AY227491 | AY227543 |
| *Aeromys tephromelas* | Ate | AY227594 | AY227482 | AY227536 |
| *Eupetaurus cinereus* | Eci1 | AY227596 | AY227484 | AY227538 |
| *Pteromys volans* | Ptv | AY227605 | AY227493 | AY227545 |
| *Pteromys volans* |  |  | JQ230001 | JQ230001 |
| *Petaurista alborufus* | Pal1 | AY227601 | AY227489 | AY227541 |
| *Petaurista yunanensis* |  |  | KX528208 | KX528208 |
| *Petaurista hainana* |  |  | JX572159 | JX572159 |
| *Eoglaucomys fimbriatus* | Efi1 | AY227597 | AY227485 | AY227562 |
| *Glaucomys volans* | Gvo | AY227598 | AY227486 | AY227559 |
| *Glaucomys volans* |  |  | AF038020 | AF038020 |
| *Hylopetes phayrei* | Hph | AY227599 | AY227487 | AY227539 |
| *Hylopetes phayrei* |  |  | KC447305 | KC447305 |
| *Hylopetes alboniger* |  |  | KX710106 | KX710106 |
| *Petaurillus kinlochii* | Pki | AY227602 | AY227490 | AY227542 |
| *Petinomys setosus* | Pse1 | AY227604 | AY227492 | AY227544 |
| *Iomys horsfieldi* | Iho6 | AY227600 | AY227488 | AY227540 |
| Outgroup |  |  |  |  |
| *Tamiasciurus hudsonicus* | Thu1 | AY227622 | AY227504 | AY227555 |
| *Ratufa bicolor* | Rbi | AY227608 | AY227496 | AY227548 |

^#^ These haplotypes were sequenced in this study.
