# Supplementary figures and images for: Taxonomic revision and phylogenetic position of the flying squirrel genus Biswamoyopterus (Mammalia, Rodentia, Sciuridae, Pteromyini) on the northern Indo-China peninsula
Source: Zookeys. 2020 Jun 9;939:65–85. doi: 10.3897/zookeys.939.31764 (PMC7341418; doi:10.3897/zookeys.939.31764)

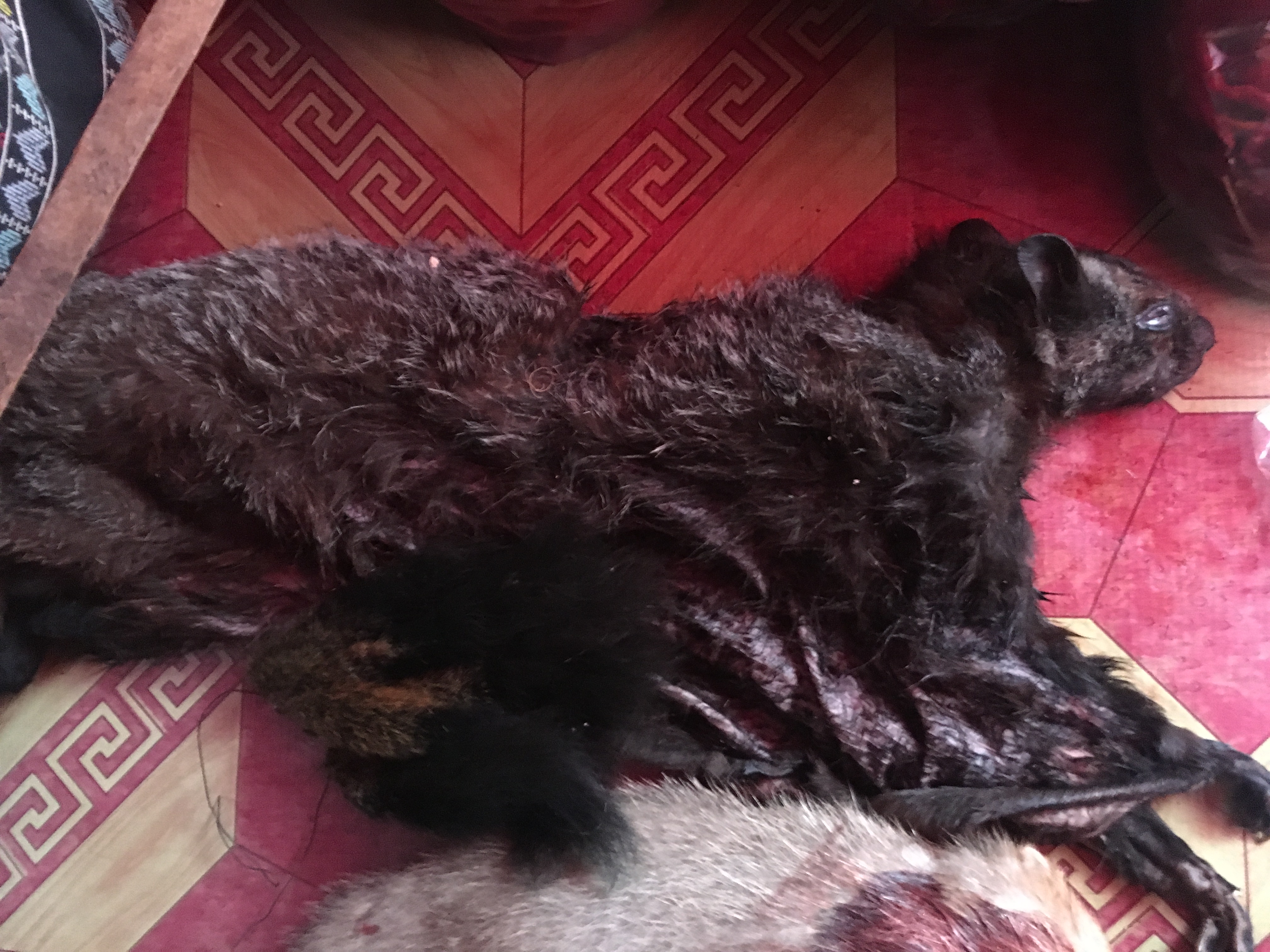

Supplement: Supplementary material 2 — Figure S1. Photograph of specimen L35 from northern Laos [file zookeys-939-065-s002.jpg]
